# Childhood traumatic experiences in people with obesity with and without eating disorders who are seeking bariatric surgery: the role of attachment relationships and family functioning

**DOI:** 10.1007/s40519-024-01638-8

**Published:** 2024-01-22

**Authors:** Emanuela Paone, Michela Di Trani, Enrico Visani, Cinzia Di Monte, Virginia Campedelli, Gianfranco Silecchia, Carlo Lai

**Affiliations:** 1 Department of Medical Surgical Sciences and Biotechnologies, Faculty of Pharmacy and Medicine, Polo Pontino, Bariatric and Metabolic Reference Centre SICOB, Sapienza University, Rome, Corso della Repubblica, 79, 04100 Latina, Italy; 2 Department of Dynamic and Clinical Psychology, and Health Studies, Faculty of Medicine and Psychology, Sapienza University of Rome, Via degli Apuli, 1, 00185 Rome, Italy; 3 Italian Institute of Relational Psychotherapy (IIPR), Viale Regina Margherita, 269, 00198 Rome, Italy; 4 Department of Medical and Surgical Sciences and Translational Medicine, Faculty of Medicine and Psychology, Bariatric Center of Excellence SICOB, Sapienza University of Rome, Via di Grottarossa 1035, 00189 Rome, Italy

**Keywords:** Obesity, Traumatic childhood experiences, Attachment, Family functioning, Eating disorders

## Abstract

**Purpose:**

The present study examines the impact of traumatic childhood experiences in people with obesity seeking bariatric surgery. It considers the presence of eating disorders (ED) in the population with obesity and tests the role of attachment and family relationships as mediators of the relationship between traumatic events and ED.

**Method:**

110 participants with severe obesity and 98 participants of a healthy weight (control group) filled out The Childhood Trauma Questionnaire (CTQ-SF), the Attachment Style Questionnaire (ASQ) and the Family Adaptability and Cohesion Evaluation Scale (FACES IV).

**Results:**

Comparing the two groups on psychological variables, higher scores in the CTQ Emotional neglect and ASQ insecure attachment scales emerged in the control group than the group with obesity. Considering the presence/absence of an ED only in the group with obesity, and comparing these subgroups, higher scores in traumatic experiences emerged in the individuals with obesity and with ED than the individuals with obesity without ED. Moreover, participants with ED scored higher in ASQ insecure attachment and had lower levels of flexibility in family functioning than the group without ED. Finally, Logistic Regression models showed that insecure anxious attachment and dysfunctional familial relationships affected the relationship between traumatic childhood experiences and the presence of ED in the group with obesity.

**Conclusion:**

These findings suggest the importance focusing on psychosocial factors linked to obesity, specifically on attachment styles and familial relationships as emotion regulation strategies, since the impact of traumatic childhood events on psychopathology could be ameliorated by an individual’s ability to rely on a significant attachment figure.

**Level of evidence:**

Level II, evidence obtained from well-designed controlled trials without randomization.

## Introduction

Obesity is a major public health concern worldwide. In recent years, the interest has increased in analyzing psychosocial risk factors that may relate to obesity development and have an impact on surgical treatment outcomes [1, 2]. Most research to date has focused on people with obesity seeking bariatric surgery to better understand their psychosocial features with the aim of structuring adequate multidisciplinary treatments.

In this field, a growing body of research has focused on the impact of traumatic childhood experiences on obesity. Childhood traumatic experiences (CTE) refer to different types of maltreatment: emotional abuse, physical abuse, emotional and physical neglect during childhood and adolescence. Studies focused on childhood traumatic experiences have established the strong association between these events and adverse health outcomes among adults [3, 4]. In fact, research suggests that exposure to traumatic events is associated with a high prevalence and incidence of overweight or later obesity [5, 6]. Moreover, Belli et al. [7] found a correlation between CTE and dissociative symptoms and binge eating disorder in obese patients.

Among bariatric surgery patients there are contrasting results: Stricklen [8] showed that childhood adversity is significantly correlated with body mass index (BMI); D’Argenio et al. [9] highlighted the association between early-life trauma and obesity in a sample of obese people with and without psychiatric disorders; Akduman et al. [10] found relation between CTE and problematic eating behaviour in a sample of obese patients waiting for bariatric surgery. While some studies found an association between childhood maltreatment and increased prevalence of lifetime mood, anxiety, and substance use disorders in this population [11], another study reported that these experiences were not significantly associated with BMI and binge eating disorder but were significantly associated with higher depression and lower self‐esteem [12]. It is not yet clear whether the relationship between CTE and BMI is mediated by psychopathology in severe obesity.

Another important psychological factor explored among individuals with obesity is attachment style, which has been found to be related to both CTE and obesity. Attachment style is a concept derived from John Bowlby’s attachment theory [13] and describes how an individual typically interacts in close, intimate relationships with “attachment figures” to which they are emotionally bonded to, like parents, children, or romantic partners [14]. Thomson and Jaque [15] demonstrated a strong relationship between increased CTE and greater attachment insecurity, but also showed that the correlation between CTE and insecure attachment is not linear: within ≥ 4 CTE group, 51.4% were classified as secure in the Adult Attachment Interview [16], which assesses an adult’s attachment style based on his or her early life experiences, particularly with caregivers. The authors suggested that despite exposure to high CTE, some psychological factors may be related to attachment security, like resilience, and college education. Still on the subject, Lin et al. [17] indicated that exposure to adverse experiences during childhood was significantly associated with higher levels of attachment anxiety, supporting the theory deeming early socioemotional experiences as important determinants of felt security and future attachment patterns in close relationships [13]. In particular, the study [17] showed that attachment anxiety measured by a self-report questionnaire designed a to assess adult attachment styles in romantic relationships, Experience in Close Relationship Scale [18] is an important mediator between CTE and the development of somatic symptoms. In fact, the association of CTE with somatic symptoms was stronger when levels of attachment anxiety were high [17]. Therefore, there is a relationship between CTE and attachment, but other factors play a role in it.

Regarding the relationship between BMI and attachment, results of a meta-analysis [19] indicated that higher BMI was associated with lower attachment security and/or higher attachment insecurity. D’Argenio et al. [9] found a link between anxious attachment and obesity; higher attachment anxiety has been associated with greater emotional eating [20] and binge eating symptoms [21].

Research has shown that attachment patterns affect regulatory behaviours, which include the regulation of sleep, emotions, and eating behaviour [22, 23]. When there is a pattern of insecure attachment, food can become an alternative to the sensations related to closeness to the caregiver, leading to the development of maladaptive eating habits and/or obesity. In this perspective, it is important to consider the role of family on the development of eating habits and obesity. A review of literature has shown that poor family functioning was associated with increased risk of obesity and overweight in children and adolescents. Obese children and adolescents were more likely to come from families with poor functioning [24]. However, there are no studies that have explored the relationship between family functioning and adult obesity.

Overall, research literature has identified a relationship between CTE and both obesity and in people seeking bariatric surgery, though this relationship is controversial and probably nonlinear. It is also unclear whether this relationship is influenced by the presence of psychopathology in the population with obesity examined. Furthermore, no study examines the complex relationship between CTE, attachment, and family relationships in individuals with obesity, even though these variables seem to be linked to obesity, both from a theoretical and an empirical point of view.

The present study examined, for the first time, the connection between CTE and obesity, considering the role of attachment and quality of family functioning, within a complex model. In a first step, with the aim to explore these variables, people with obesity seeking bariatric surgery were compared to the general population. In a second step, the hypothesis that CTE are specifically linked to the presence of an eating disorder in the population with obesity has been tested, and the role of attachment and family relationships in this relationship has been evaluated.

## Materials and methods

### Procedures

Participants were divided into two groups: group with obesity and control group. The outpatients with severe obesity were recruited during the psychological assessment for eligibility for bariatric surgery at the Centre of Bariatric Excellence at “Sapienza” University of Rome (Italy). Patients were volunteers, assessed between March 2019 and October 2019.

Inclusion criteria to be selected for the study were between the ages of 18 and 60 years old and being of Italian nationality. Exclusion criteria were presence of severe psychiatric illness requiring acute care (i.e., major depression, psychotic symptoms) and lower education level than middle school. Moreover, patients undergoing revision surgery were excluded from this study to avoid potential confounding effects.

The procedure for the psychological assessment of access to bariatric surgery is carried out according to the suggestions for psychological-psychiatric procedures in obesity surgery of the Italian Society of Obesity Surgery and Metabolic Diseases (SICOb) [25], through the clinical interview that includes: examination of the current and previous mental state, analysis of eating behaviour and weight history, analysis of motivation, psychosocial aspects and the ability to adhere to the post-intervention programme [25].

During the analysis of eating behaviour, dysfunctional eating behaviour is identified that is linked to a lower or higher psychopathological intensity related to dysregulation of the emotional system [25]. In this case, the clinical psychologist assesses the presence of binge eating symptoms and if these are highlighted, a specialist assessment by the psychiatrist is made. In our study, participants who showed a possible presence of binge eating disorder were assessed by the psychiatrist who confirmed the diagnosis of binge eating disorder according to the DSM 5 criteria [26].

Different procedures were used to recruit the control group. This comprised individuals who were not obese recruited voluntarily through a survey online created especially for this research with Google Forms, a tool that can create surveys in the web browser. The self-report questionnaires administered in the study were included in this survey. Inclusion and exclusion criteria were the same as the group with obesity, but in addition individuals with a BMI greater than 30 were excluded from the control group. The period of recruitment was the same as that of the group with obesity.

This study was performed in line with the principles of the Declaration of Helsinki and written informed consent was obtained by all participants. The study protocol was approved by Ethics Committee of University Department of Dynamic, Clinical Psychology and Health Studies of “Sapienza” University of Rome (01/20/2020, No. 0000078).

### Measures

For the present study, four self-report questionnaires exploring different areas were assessed:

- A *socio-demographic questionnaire*, including information about self-reported age, sex, weight, height, psychiatric and physical disorders, educational level, work status and information about the participant’s family.
- The *Childhood Trauma Questionnaire—Short Form CTQ-SF* [27] a self-report instrument that retrospectively assesses different types of maltreatment during childhood and adolescence. These are: emotional abuse, physical abuse, emotional and physical neglect. The CTQ contains 28 items and is rated on a 5-point Likert scale (from 1 = never to 5 = very often). The total score considers the severity of multiple forms of abuse and neglect. In the present study, the Italian translation of this scale was used [28] and internal consistency was acceptable with a Cronbach’s *α* of 0.78.
- The *Family Adaptability and Cohesion Evaluation Scale*—*FACES IV* [29], a family self-report assessment designed to assess family cohesion and family flexibility which are the two central dimensions of the Circumplex Model of Marital and Family Systems. The FACES IV contain 62 items rated on a 5-point Likert scale (from 1 = totally disagree to 5 = totally agree) and has two balanced subscales (cohesion and flexibility) and four unbalanced subscales (disengaged, enmeshed, rigid, and chaotic). Additionally, FACES IV also has subscales for family communication and family satisfaction. The typical scoring method is to create ratio scores for both the Cohesion and Flexibility scales. In the present study, we used the translation and validation Italian versions on this scale [30]. The reliability coefficients of internal consistency were acceptable for this study (Cronbach’s *α* values = 0.84).
- The *Attachment Style Questionnaire—ASQ* [31] is a 40-item questionnaire. The ASQ is composed of five scales: “confidence in self and others”, that assesses an individual's self-assurance and comfort in close relationships; “discomfort with closeness”, that measures the level of unease someone experiences in emotionally close relationships; “relationships as secondary”, that evaluates the degree to which a person prioritizes independence and self-sufficiency over being involved in close relationships; “need for approval”, that assesses the extent to which an individual seeks approval from their partner in a relationship; and preoccupation with relationships, that measures the level of preoccupation a person might have with their romantic relationships. Items were scored on a 6-point Likert-type scale ranging from “totally disagree” to “totally agree”. We used the translation and validation Italian versions of this scale [32]. The internal consistency was good with a Cronbach’s *α* of 0.85.

### Statistical analyses

The statistical analyses were conducted using the Statistical Package for Social Science (SPSS) version 25 for Windows (IBM, Armonk, NY, USA). Demographic characteristics of the group with obesity versus the control group were compared using *χ*^2^ analysis for categorical variables and one-way ANOVAs for continuous variables.

In order to compare individuals with obesity and control group on traumatic experiences, attachment features and family functioning, one-way ANOVAs were also performed. Moreover, participants with obesity and a diagnosis of an eating disorder and participants with obesity without eating disorders were compared on the same variables, by another series of one-way ANOVAs.

Finally, a set of logistic regression models (logit), including the presence/absence of eating disorder (ED) in the group with obesity as a dependent variable, were carried out to evaluate the association between traumatic experiences and ED in patients with obesity, including attachment features and family functioning variables as count.

## Results

A total of 208 participants were enrolled between March 2019 and October 2019. One hundred and ten people were part of the group with obesity and were predominantly female (*n* = 92; 83.6) with a mean age of 44.45 years (SD = 11.38). The control group, on the other hand, consisted of 98 participants with a female predominance (*n* = 72; 73.5%) and a mean age of 38.72 years (SD = 12.88). Table 1 shows the participants’ socio-demographic characteristics.

**Table 1:** Socio-demographic characteristics of participants (*n* = 208)

	Group with obesity (*n* = 110)			Control group (*n* = 98)		
	*n* (%)	Mean	sd	*n* (%)	Mean	sd
Weight		111.23 kg	20.68		67.03 kg	12.69
Body mass index (BMI)		41.43	6.31		23.66	3.29
Underweight (16.0–18.5)				2 (2.04%)		
Healthy weight (18.5–24.9)				64 (65.31%)		
Overweight (25.0–30.0)				32 (32.65%)		
Obese Class I (30.0–34.9)	13 (11.82%)					
Obese Class II (35.0–39.9)	41 (37.27%)					
Obese Class III (> 40.0)	56 (50.91%)					
Psychiatric disorders						
Not present	49 (45.22%)					
Mild anxiety symptoms	7 (6.36%)					
Mild depression	20 (18.12%)					
Binge eating disorder	34 (30.30%)					
Education						
Secondary education	39 (35.45%)			1 (1.02%)		
Higher education	51 (46.37%)			23 (23.46%)		
Professional course	11 (10.00%)			2 (2.04%)		
University	9 (8.18%)			72 (73.48%)		
Work						
Student	2 (1.81%)			16 (16.33%)		
Looking for work	1 (0.90%)			7 (7.14%)		
Employed	52 (47.28%)			65 (66.33%)		
Unemployed	23 (20.91%)			4 (4.08%)		
Housewife	21 (19.10%)			3 (3.06%)		
Retired	4 (3.64%)			3 (3.06%)		
Other	7 (6.36%)					
Marital status						
Single	12 (10.92%)			24 (24.27%)		
Married	66 (60.00%)			39 (39.30%)		
Separated	13 (11.82%)			6 (6.12%)		
Cohabitation	18 (16.36%)			21 (21.13%)		
Widowed	1 (0.90%)					
Other				9 (9.18%)		
Actual family role						
Father/husband	18 (16.36%)			10 (10.20%)		
Mother/wife	72 (65.46%)			32 (32.66%)		
Son	10 (9.09%)			40 (40.82%)		
Other	10 (9.09%)			16 (16.32%)		
Type of family						
Couple	69 (62.74%)			54 (55.10%)		
Two parents (biological family)	23 (20.92%)			28 (28.58%)		
Two parents (reconstructed family)	1 (0.90%)			3 (3.06%)		
Two parents (adoptive family)	1 (0.90%)			6 (6.12%)		
One parent	6 (5.45%)			7 (7.14%)		
Other	10 (9.09%)					
Cohabitation						
Only	8 (7.27%)			11 (11.22%)		
With parents	10 (9.09%)			22 (22.44%)		
With partner	21 (18.09%)			26 (26.54%)		
With others				3 (3.06%)		
With children	11 (10.00%)			2 (2.04%)		
With spouse and children	55 (50.00%)			27 (27.56%)		
Other	5 (5.55%)			7 (7.14%)		

No differences emerged between the two groups on age (*F* = 3.58; *p* = 0.06) and gender (*χ*^2^ = 3.83; *p* = 0.07), whereas a significant difference was found on education (*χ*^2^ = 104.09; *p* = 0.00), showing a lower level of education in the obese group than the control group.

Differences between participants with obesity and control groups on traumatic experiences, attachment features and family functioning are reported in Table 2. Regarding traumatic experiences, results showed a higher level of CTQ Emotional Neglect in the control group (*p* = 0.00), which also showed higher level of insecure attachment (ASQ Discomfort with Closeness *p* = 0.00; Need for Approval *p* = 0.01; Preoccupation with Relationships *p* = 0.00) and a lower level of secure attachment (ASQ Confidence *p* = 0.00) than the group with obesity. No differences between the two groups were found with respect to family functioning.

**Table 2:** One-way ANOVAs between group with obesity and control group on traumatic experiences, attachment features and family functioning

	Group with obesity		Control group		*F*	*p*
	Mean	sd	Mean	sd		
CTQ-SF^a^ emotional neglect	16.50	3.70	18.92	4.39	4.10	**0.04**
CTQ-SF^a^ sexual abuse	7.07	3.60	7.29	3.17	0.21	0.65
CTQ-SF^a^ physical abuse	7.78	2.42	8.08	2.67	0.64	0.43
ASQ^b^ confidence	33.81	5.57	30.72	5.16	16.03	**0.00**
ASQ^b^ discomfort with closeness	33.68	7.64	37.54	8.28	11.44	**0.00**
ASQ^b^ relationships as secondary	14.95	5.98	15.40	5.11	0.32	0.58
ASQ^b^ need for approval	17.76	7.04	20.42	7.51	6.48	**0.01**
ASQ^b^ preoccupation with relationships	23.42	7.95	27.69	7.95	16.72	**0.00**
FACES-IV^c^ cohesion	28.61	4.43	27.99	4.81	0.87	0.35
FACES-IV^c^ flexibility	26.76	4.38	25.90	5.18	1.58	0.21
FACES-IV^c^ disengaged	15.92	5.47	16.24	5.38	0.17	0.69
FACES-IV^c^ enmeshed	16.47	4.71	17.30	4.93	1.45	0.23
FACES-IV^c^ rigid	19.35	3.85	18.50	4.51	2.02	0.16
FACES-IV^c^ chaotic	15.81	5.50	14.97	5.35	1.17	0.28
FACES-IV^c^ communication	39.31	8.17	37.35	8.8	2.59	0.10
FACES-IV^c^ satisfaction	36.14	9.00	37.42	8.9	0.99	0.31

Values in bold indicate statistical significance at the *p* < 0.05 level

^a^Children trauma questionnaire—short form

^b^Attachment style questionnaire

^c^Family adaptability and cohesion scale-V version

Based on the unexpected results of the previous comparison, specific characteristics of the group with obesity were explored, considering the presence/absence of an eating disorder diagnosis. Specifically, the participants with obesity were split into obese participants with an eating disorder (ED with obesity, *N* = 34) and participants with obesity without an eating disorder (non-ED with obesity, *N* = 76), and the two groups were compared on traumatic experiences, attachment features and family functioning.

Table 3 includes the results of the one-way ANOVAs, showing higher levels of CTQ Emotional Neglect (*p* = 0.04), Sexual (*p* = 0.00) and Physical Abuse (*p* = 0.00) in the group with eating disorder, than participants with obesity without eating disorder. Regarding attachment features, participants with eating disorder showed higher levels of ASQ Need for Approval (*p* = 0.00) and Preoccupation with Relationships (*p* = 0.02). Finally, lower levels of FACES-IV Flexibility (*p* = 0.02), Communication (*p* = 0.02), and Satisfaction (*p* = 0.01) emerged in the group with eating disorder than in the group without eating disorder.

**Table 3:** One-way ANOVAs between individuals with obesity and with a diagnosis of eating disorder and individuals with obesity without a diagnosis of eating disorder on traumatic experiences, attachment features and family functioning

	ED group with obesity		Non-ED group with obesity		*F*	*p*
	Mean	sd	Mean	sd		
CTQ-SF^a^ emotional neglect	19.78	10.32	15.00	6.28	8.32	**0.00**
CTQ-SF^a^ sexual abuse	8.78	5.70	6.29	1.54	11.48	**0.00**
CTQ-SF^a^ physical abuse	8.78	3.92	7.33	1.02	8.30	**0.00**
ASQ^b^ confidence	33.16	5.59	33.99	5.50	0.49	0.48
ASQ^b^ discomfort with closeness	35.09	7.41	33.03	7.76	1.59	0.21
ASQ^b^ relationships as secondary	15.50	5.78	14.74	6.12	0.35	0.55
ASQ^b^ need for approval	21.16	7.56	16.28	6.27	11.59	**0.00**
ASQ^b^ preoccupation with relationships	26.16	7.75	22.23	7.82	5.54	**0.02**
FACES-IV^c^ cohesion	27.44	4.92	29.10	4.13	3.14	0.08
FACES-IV^c^ flexibility	25.28	5.22	27.36	3.75	5.20	**0.02**
FACES-IV^c^ disengaged	17.34	4.95	15.32	5.63	3.04	0.08
FACES-IV^c^ enmeshed	17.66	4.37	15.99	4.79	2.80	0.10
FACES-IV^c^ rigid	19.50	3.39	19.28	4.10	0.07	0.79
FACES-IV^c^ chaotic	17.13	5.52	15.22	5.47	2.64	0.11
FACES-IV^c^ communication	36.59	9.85	40.56	7.01	5.38	**0.02**
FACES-IV^c^ satisfaction	32.91	9.31	37.61	8.52	6.32	**0.01**

Values in bold indicate statistical significance at the *p* < 0.05 level

^a^Children trauma questionnaire—short form

^b^Attachment style questionnaire

^c^Family adaptability and cohesion scale-V version

As shown in Table 4, the logistic regression with CTQ scales as predictor and ED diagnoses as outcome did not show significant associations between abuse variables and presence of eating disorder (*p* > 0.05). Moreover, we inserted the ASQ and FACES-IV dimensions that showed a significant difference in the ANOVAs between participants with obesity and with eating disorders and participants with obesity without eating disorders into the logistic regression model as count (respectively: Need for Approval and Preoccupation with Relationships; Flexibility, Communication and Satisfaction).

**Table 4:** Logistic regression (logit) analysis on the associations between traumatic experiences in childhood as predictors and the presence of eating disorders as outcome in the group with obesity without (a) and with (b) and (c) inserting the attachment and (d), (e), and (f) family dimensions as count

	Estimate	Standard error	Wald test	*p*
(a) Without attachment dimensions as count				
Intercept	3.22	1.17	7.6	0.006
CTQ-SF^a^ emotional neglect	− 0.05	0.04	1.8	0.179
CTQ-SF^a^ sexual abuse	− 0.20	0.10	3.7	0.055
CTQ-SF^a^ physical abuse	− 0.02	0.20	0.1	0.900
(b) With need for approval as count				
Intercept	3.17	0.30	110.9	< 0.0001
CTQ-SF^a^ emotional neglect	− 0.02	0.01	8.4	**0.004**
CTQ-SF^a^ sexual abuse	− 0.19	0.02	69.8	**< 0.0001**
CTQ-SF^a^ physical abuse	− 0.11	0.05	5.1	**0.024**
(c) With preoccupation with relationships as count				
Intercept	3.49	0.29	145.5	< 0.0001
CTQ-SF^a^ emotional neglect	− 0.04	0.01	24.7	**< 0.0001**
CTQ-SF^a^ sexual abuse	− 0.25	0.02	105.5	**< 0.0001**
CTQ-SF^a^ physical abuse	− 0.07	0.04	2.2	0.133
(d) With flexibility as count				
Intercept	3.24	0.24	183.2	< 0.0001
CTQ-SF^a^ emotional neglect	− 0.05	0.01	41.9	**< 0.0001**
CTQ-SF^a^ sexual abuse	− 0.22	0.02	98.1	**< 0.0001**
CTQ-SF^a^ physical abuse	− 0.01	0.04	0.1	0.805
(e) With communication as count				
Intercept	3.43	0.20	295.6	< 0.0001
CTQ-SF^a^ emotional neglect	− 0.05	0.01	79.4	**< 0.0001**
CTQ-SF^a^ sexual abuse	− 0.19	0.02	123.1	**< 0.0001**
CTQ-SF^a^ physical abuse	− 0.05	0.03	2.3	0.125
(f) With satisfaction as count				
Intercept	3.27	0.20	268.6	< 0.0001
CTQ-SF^a^ emotional neglect	− 0.05	0.01	70.4	**< 0.0001**
CTQ-SF^a^ sexual abuse	− 0.18	0.02	99.9	**< 0.0001**
CTQ-SF^a^ physical abuse	− 0.032	0.03	0.8	0.355

Values in bold indicate statistical significance at the *p* < 0.05 level

^a^Children trauma questionnaire—short form

When the ASQ scales and FACES-IV variables were inserted as count in the regression, the associations between the emotional and sexual scales of CTQ and ED diagnoses became significant. The association between physical abuse and ED diagnosis was significant only when the Need for Approval was inserted as count in the logistic regression model.

## Discussion

This is the first study examining the relationship between childhood traumatic experiences (CTE), the role of attachment, and family functioning in people with obesity. Furthermore, the hypothesis that CTE are linked to the presence of an eating disorder in the population with obesity has been tested, and the role of attachment and family relationships in this relationship has been evaluated.

Statistical analysis showed no differences in socio-demographic features between individuals with obesity and control group. The two groups differ only for their educational level; in fact, according to the literature [33], the control group had a higher education level. Furthermore, the two groups also differed depending on adverse childhood experiences and attachment style. Contrary to what was expected, one-way ANOVAs indicated that non-obese participants reported a greater number of traumatic experiences in childhood, in particular experiences of emotional neglect, and more insecure attachment than participants with obesity. According to previous research, the relationship between childhood traumatic experiences and obesity [8, 9], and between attachment and obesity are controversial [9, 19], but some studies have reported a large percentage of sexual abuse [12, 34] and maltreatment [35] in people with obesity who are candidates for bariatric surgery. We can assume that the bias due to the exclusion of people with obesity and with severe psychiatric disorders (i.e. psychosis and major depressive disorders) influenced these results, indeed experiences of child maltreatment are usually associated with a greater risk of developing psychopathologies [12], and of insecure attachment in adulthood [15, 17].

Many studies have reported that individuals with obesity who are candidates for bariatric surgery are highly vulnerable to developing a psychiatric disorder [36, 37]. Therefore, the relationship between childhood traumatic experiences and obesity is possibly mediated by the presence of psychiatric disorder in people with obesity. This hypothesis is reinforced by the results obtained from the comparison between participants with obesity and ED and participants with obesity without ED: the findings of one-way ANOVAs between these two groups of patients with obesity showed that the presence of an ED is associated with a major presence of emotional neglect, physical, and sexual abuse. Regarding attachment, individuals with obesity and with ED showed more insecure attachment, in particular anxious dimensions, suggesting a relationship functioning characterized by emotional dysregulation. Consistently with our results, literature has specifically linked anxious attachment with obesity and eating disorders, possibly through the tendency to engage in disinhibited eating and to seek comfort through overeating [38].

With regard to family functioning, our participants in the ED group with obesity showed lower capability to manage roles, rules, and negotiations (Flexibility), lower Communication levels, defined as the positive skills utilized in the couple or family system that supports levels of familiar flexibility, and lower Satisfaction levels, than group without eating disorder.

Moreover, logistic regression analysis revealed that attachment and family relational dimensions affected relationship between the ED and CTE: traumatic emotional and sexual experiences in childhood predicted the presence of ED in participants with obesity only when insecure attachment dimensions (Need for Approval and Preoccupation with Relationships) and family relational dimensions (Flexibility, Communication, and satisfaction) were inserted as count in this relationship. Indeed, physical abuse was associated with the presence of ED in participants with obesity only when the Need for Approval attachment dimension was inserted as count.

These findings suggest focusing attention on a set of psychosocial factors which are linked to obesity and show an important role of familial relationships and functioning. The impact of adverse events in one's childhood could be ameliorated by the possibility of relying on a significant other and functional regulation strategies learned within the attachment relationship. In particular, the effect of childhood traumatic events on eating psychopathology is an area that has generated considerable interest and debate in the literature showing the importance of considering potential mediators and creating a more complex model, especially for the effect of childhood emotional abuse [39]. In particular, our finding shows anxious attachment seems to have an important role; it could be a risk factor in the onset of eating disorders.

Clinical implications of our findings concern the role of psychology in a multidisciplinary team for obesity. If clinicians can understand and consider during their diagnostic process how psychological features impact the development of obesity, they may be better equipped to plan and build comprehensive treatments for these patients. A specific focus on attachment relationships, especially for patients with obesity and with traumatic history and/or psychiatric disorders could be a powerful intervention in supporting bariatric surgery.

The strength of this study was to assess family functioning in people with obesity, with and without an eating disorder, compared to people with a healthy weight. In fact, to our knowledge, this is the first study that assesses the role of attachment, and family functioning in the relationship between childhood traumatic experiences and obesity. However, this study has some limitations. First, the group with obesity and the control group were recruited in different contexts and the assessment was carried out in two different ways. Another limitation is to be found in the inclusion criteria with the wide age range (18–60). The decision to include a wide age range was made in order to obtain more complete and representative data of the general population suffering from obesity, thus improving the external validity of the study. Future studies could consider a narrower age range to understand how variables unfold at different ages.

Last limitation is the use of only self-report measures. It is also important to consider that these instruments, in the group with obesity, were administered during the process to assess eligibility for bariatric surgery. This context could have amplified the known phenomenon of social desirability, already highlighted in the research for individuals who are obese [40, 41]. Future research should include the use of different instruments to assess these variables in the population with obesity. Identifying and managing social desirability through the administration of specific questionnaires, such as the Marlowe–Crowne Social Desirability Scale (MC-SDS) [42] or the Paulhus Deception Scales (PDS) [43], can help to obtain more authentic and reliable information from study participants. Lastly, weight and height could be collected with objective instruments.

### What is already known on this subject?

Research suggests that exposure to traumatic events is associated with obesity and in people seeking bariatric surgery, though this relationship is controversial and probably nonlinear. Indeed, it is unclear whether this may be influenced by psychosocial factors such as the presence of psychopathology, attachment style or family relationships in individuals with obesity.

### What does this study add?

The present study suggests that the impact of adverse events in one’s childhood could be ameliorated by the possibility of relying on a significant other and functional regulation strategies learned within the attachment relationship in people with obesity that are undergoing bariatric surgery, with and without an eating disorder, compared to people with a healthy weight. Moreover, our finding shows anxious attachment seems to have an important role and it could be a risk factor in the onset of eating disorders.

## Data Availability

The datasets generated during and/or analysed during the current study are available from the corresponding author on reasonable request.
